# Open-source magnetic resonance imaging acquisition: Data and documentation for two validated pulse sequences

**DOI:** 10.1016/j.dib.2022.108105

**Published:** 2022-03-28

**Authors:** Gehua Tong, Andreia S. Gaspar, Enlin Qian, Keerthi Sravan Ravi, John Thomas Vaughan, Rita G. Nunes, Sairam Geethanath

**Affiliations:** a Department of Biomedical Engineering, Columbia University in the City of New York, New York, NY, USA; b Columbia Magnetic Resonance Research Center, Columbia University in the City of New York, New York, NY, USA; c Institute for Systems and Robotics (ISR-Lisboa)/LaRSyS and Department of Bioengineering, Instituto Superior Técnico, Universidade de Lisboa, Lisbon, Portugal; d Department of Radiology, Columbia University Irving Medical Center, Columbia University in the City of New York, New York, NY, USA

**Keywords:** Repeatability, Pulse sequence programming, MRI simulation, Reproducible research, T_1_/T_2_ mapping

## Abstract

Raw data, simulated and acquired phantom images, and quantitative longitudinal and transverse relaxation times (T_1_/T_2_) maps from two open-source Magnetic Resonance Imaging (MRI) pulse sequences are presented in this dataset along with corresponding “.seq” files, sequence implementation scripts, and reconstruction/analysis scripts [1]. Real MRI data were collected from a 3T Siemens Prisma Fit and a 1.5T Siemens Aera via the Pulseq open-source MR sequence platform, and corresponding in silico data were generated using the simulation module of Virtual Scanner [2]. This dataset and its associated code can be used to validate the pipeline for using the same pulse sequences at other research sites using Pulseq, to provide guidelines for documenting and sharing open-source pulse sequences in general, and to demonstrate practical, customizable acquisition scripts using the PyPulseq library.

## Specifications Table

**Table:** 

Subject	Medical Imaging
Specific subject area	Magnetic Resonance Imaging (MRI) pulse sequence design and documentation using open-source, multi-vendor programming platforms.
Type of data	Code (.m, .ipynb) Sequence file (.seq) Raw data (.mat) Image (.mat) Figure (.png) Table (.xlsx) Form (.pdf)
How the data were acquired	Raw MRI data were acquired on a Siemens 3T Prisma Fit system (“main site” or “developer”) and a Siemens 1.5T Aera system (“second site” or “user”). The Pulseq 1.2.1 (second site: 1.3.1) interpreter was used [3]. All sequences (.seq) data were generated from Google Colab notebooks (.ipynb) in Python using PyPulseq (version 1.2.0) [4]. For all qualitative scans, the American College of Radiology (ACR) large MRI phantom [5] was acquired. Quantitative scans used the T_1_ and T_2_ planes of the International Society for Magnetic Resonance Medicine / National Institute of Standards and Technology (ISMRM/NIST) phantom [6] for T_1_ and T_2_ mapping, respectively. Reconstruction scripts were provided in MATLAB (.m). The PDF forms were created using a combination of Adobe Indesign and Adobe Acrobat DC and filled by the two scanning sites. Experimental parameters are shown in Table 1.
Data format	Simulated Raw Filtered Analyzed
Description of data collection	Images from the same tested sequence were acquired in the same session at each site. Multi-slice or multi-contrast images were normalized across all slices or contrasts to the range [0,1] after channel combination.
Data source location	The developer side data: • Institution: Columbia University in the City of New York • City: New York, NY 10,027 • Country: United States • Latitude and longitude for collected samples/data: 40°49′01.0″N 73°57′28.3″W (GPS: 40.816952534117384, −73.9578507140652) The user side data: • Institution: Universidade de Lisboa • City: Lisbon • Country: Portugal • Latitude and longitude for collected samples/data: 38°45′22.1″N 9°11′33.2″W (GPS: 38.756134666694756, −9.19254883734996)
Data accessibility	Repository name: Mendeley Data Data identification number: (DOI: 10.17632/8458pz722c.5) Direct URL to data: https://data.mendeley.com/datasets/8458pz722c/5
Related research article	G. Tong, A.S. Gaspar, E. Qian, K.S. Ravi, J.T. Vaughan, R.G. Nunes, S. Geethanath, A framework for validating open-source pulse sequences, Magn. Reson. Imaging. 87 (2022) 7–18. https://doi.org/10.1016/j.mri.2021.11.014.

## Value of the Data

• The packaged and documented sequence waveforms, raw data, and reconstructed images can serve as a baseline for future efforts in standardizing open-source MRI acquisition, reconstruction and mapping pipelines.
• Researchers working on repeatability and cross-site studies can use the data as a starting point for communicating and harmonizing sequences using the Pulseq platform.
• Students working in MR can follow the framework to gain practical knowledge in designing MR sequences.
• Further experiments can use the programs in vivo to evaluate clinical usefulness of open-source pulse sequences.
• The sequence scripts can be used to assess the optimal degrees of freedom for sequence parameter customization. That is, how many changes and options should a sequence script allow before the more efficient choice is to branch out into multiple scripts.
• Efforts in Accessible MRI can use the data, acquired using open-source sequences on major commercial scanners, as a reference point for experiments using the same sequences on new hardware.

## 1 Data Description

See Table 2 for the list of MRI terminology abbreviations for this dataset.

### 1.1 Main site

#### 1.1.1 Qualitative irse

In the main folder (developer_main_site/IRSE), the sequence implementation is presented in a Jupyter notebook (write_irse_interleaved_split_grad.ipynb). In addition, a developer PDF form (IRSE_DEV_QUALITATIVE.pdf) includes information on the test experiment including sequence parameters, hardware setup, example image, image quality measures, and safety metrics.

The simulation information (developer_main_site/IRSE/sim) provides phantom models (phantom_T1plane.mat, phantom_grid.mat) where the map dimensions are the “x” and “y'' spatial indices and simulated k-space signals (irse32_T1plane.mat, irse64_grid128.mat) with dimensions “k_x_'' and “k_y_”. Reconstructed images are included as figures (irse32_T1plane.png, irse64_grid128.png).

The acquisition folder (developer_main_site/IRSE/acq) includes the tested sequence file (irse_pypulseq_colab_256_TI150_neg4.4.seq), the randomized slice order (irse_sl_order.mat), and raw data from two repetitions from the same sequence (irse_qual_raw_data_rep1.mat, irse_qual_raw_data_rep2.mat) where the data dimensions are “samples”, “channels”, and “readouts”, in that order. In addition, the acquisition details are listed in a sheet (irse_acq_info.xlsx).

In the reconstruction folder (developer_main_site/IRSE/recon), we include the reconstruction script (reconstruct_images.m) as well as the reconstructed images (images_combined.mat) and montage figure (irse_pulseq_montage.png). Image quality metrics (irse_metrics.mat) and ACR test results (ACR_METRICS_IRSE.xlsx) [5] are presented as well.

#### 1.1.2 Qualitative TSE

Similar to 1.1.1, we present in the main folder (developer_main_site/TSE) the sequence implementation (write_tse.ipynb) and the developer PDF form (TSE_DEV_QUALITATIVE.pdf). The simulation folder (developer_main_site/TSE/sim) includes equivalent phantoms (phantom_T2plane.mat, phantom_grid.mat), simulated images (tse32_T2plane.mat, tse64_grid128.mat) and figures (tse32_T2plane.png, tse64_grid128.png). In addition to the sequence file (tse_ms_TR3000ms_TE50ms_4echoes.seq), the raw data (tse_qual_raw_data_rep1.mat, tse_qual_raw_data_rep2.mat), and the acquisition details (tse_acq_info.xlsx), the acquisition folder (developer_main_site/TSE/acq) also includes the multi-echo phase encoding order (tse_pe_info.mat) with dimensions “number of echoes” and “number of excitations”. Lastly, the reconstruction folder (developer_main_site/TSE/recon) covers the equivalent files as in 1.1.1 (reconstruct_images.m, images_combined.mat, tse_pulseq_montage.png, tse_metrics.mat, ACR_METRICS_TSE.xlsx).

#### 1.1.3 IRSE T_1_ mapping

The sequence implementation (write_irse_interleaved_split_grad.ipynb) and documentation PDF (IRSE_DEV_QUANTITATIVE.pdf) are provided like above. Acquisition data (developer_main_site/IRSE_T1_mapping/acq) includes ten sequence files (irse_T1_TI50.seq, …, irse_T1_TI3000.seq) with different inversion times (see Table 1) used to generate the T_1_ map and the acquisition details (irse_t1_acq_info.xlsx). Raw data is provided as ten separate files at different Tis (T1_mapping_raw_TI50ms.mat, etc.).

**Table 1:** Acquisition experiments and parameters. Shared parameters include: Field-Of-View (FOV): 250 mm. N: matrix size; N_s_: number of slices; thk: slice thickness; TR: repetition time; TE: echo time; TI: inversion time; FA: flip angle.

Sequence	Phantom	N	N_s_	Slice orientation	thk (mm)	TR (ms)	TE (ms)	TI (ms)	FA (degrees)
IRSE	ACR	256	11	Axial	5	2000	12	150	90
TSE	ACR	256	11	Axial	5	3000	50	None	180
IRSE T1 mapping	NIST/ISMRM T1 plane	128	1	Sagittal	6	4500	10	Multiple⁎	90,180
TSE T2 mapping	NIST/ISMRM T2 plane	128	1	Sagittal	6	4500	Multiple⁎⁎	None	90,180

⁎ TI = 50, 75, 100, 125, 150, 250, 1000, 1500, 2000, 3000 ms.

⁎⁎ TE = 7 to 161 ms (23 TEs spaced 7 ms apart).

**Table 2:** Abbreviations of MRI terminology used in this dataset.

Abbreviation	Full Terminology
MRI	Magnetic Resonance Imaging
T_1_	Longitudinal Relaxation Time
T_2_	Transverse Relaxation Time
TR	Repetition Time
TE	Echo Time
TI	Inversion Time
IRSE	Inversion Recovery Spin Echo
TSE	Turbo Spin Echo
RF	Radiofrequency
SAR	Specific Absorption Rate
PNS	Peripheral Nerve Stimulation
PSNR	Peak Signal-to-Noise Ratio
SSIM	Structural Similarity Index Measure

The reconstruction folder (developer_main_site/IRSE_T1_mapping/recon) includes a reconstruction script (reconstruct_T1_mapping_images.m), the original and low-pass filtered reconstructed images (T1_mapping_images_original.mat, T1_mapping_images_filtered.mat), and the montage of all ten filtered images (T1_mapping_images_filtered_montage.png).

The analysis data (developer_main_site/IRSE_T1_mapping/ana) includes the T_1_ mapping MATLAB code: the function for creating a regression curve following the T_1_ signal model (createFitT1.m) and the script that takes in filtered images and generates a T_1_ map (T1Mapping.m). The output map is given in units of seconds in two versions, generated from either the original or the filtered T_1_ images (T1_map.mat, T1_map_filtered.mat). The filtered image is shown in a figure after adjusting the display to eliminate the phantom background (T1_map_cleaned.png). Region-Of-Interest (ROI) values for the individual T_1_ spheres are given in a separate file (t1_by_sphere_filtered.mat).

#### 1.1.4 TSE T_2_ mapping

Similar documentation (TSE_DEV_QUANTITATIVE.pdf) and sequence notebook (write_tse_t2_mapping.ipynb) are included in the main folder (developer_main_site/TSE_T2_mapping). Acquisition data includes the single multi-echo TSE sequence with 23 echoes for variable TE T_2_ mapping (tse_multiecho_NIST_t2_sag.seq), the acquisition details (tse_t2_acq_info.xlsx), and the raw data (T2_mapping_raw_data.mat). In the reconstruction folder, equivalent script, data, and figure are provided of the 23 T_2_ mapping images, each reconstructed from samples at the same echo number and therefore the same TE (reconstruct_T2_mapping_images.m, T2_mapping_images_original.mat, T2_mapping_images_filtered.mat, T2_mapping_images_filtered_montage.png). The analysis folder contains analogous mapping scripts (createFitT2.m, T2Mapping.m), two T_2_ maps generated from original and filtered data (T2_map.mat, T2_map_filtered_TEs_5–23.mat) where the latter uses only the 5th to 23rd echoes to best capture the most T_2_ values imaged, the cleaned T_2_ map figure (T2_map_cleaned.png), and the ROI statistics (T2_by_sphere_filtered.mat).

### 1.2 Second site

#### 1.2.1 Qualitative IRSE

The “IRSE_ACR” folder contains the raw data (raw_data_irse_second_site.mat), the image montage (IRSE_images_second_site.png), and the filled user form documenting the steps performed and user feedback (IRSE_USER_QUALITATIVE.pdf).

#### 1.2.2 Qualitative TSE

The “TSE_ACR” folder contains the equivalent raw data (raw_data_tse_second_site.mat), image figure (TSE_images_second_site.png), and user form (TSE_USER_QUALITATIVE.pdf). It also provides the phase encoding order (pe_info.mat).

#### 1.2.3 IRSE T_1_ mapping

Eight raw data files are provided for the different TIs (IRSE_T1mapping_TI50_second_site.mat, …, IRSE_T1mapping_TI2000_second_site.mat). The reconstructed images and T_1_ map (T1_mapping_images_second_site.mat, T1map_second_site.png) as well as the user form (IRSE_USER_QUANTITATIVE.pdf) are included.

#### 1.2.4 TSE T_2_ mapping

Similarly, the raw data (TSE_T2mapping_t2sph_second_site.mat) and reconstructed images (T2_mapping_images_second_site.mat) are included, in addition to the map figure (T2map_second_site.png) and the user form (TSE_USER_QUANTITATIVE.pdf).

### 1.3 Documentation forms

The folder (documentation_templates) contains the empty developer and user forms (seq_validation_form_DEVELOPER.pdf, seq_validation_form_USER.pdf) for documenting test experiments in the proposed sequence validation framework [1].

## 2 Experimental Design, Materials and Methods

### 2.1 Sequence implementation

Two classic MRI sequences were implemented in the multi-vendor, open-source Pulseq format [3] in Python with the PyPulseq library [4]: Inversion Recovery Spin Echo (IRSE) and Turbo Spin Echo (TSE) [7]. The scripts were programmed in Google Colaboratory [8] as Jupyter Notebooks.

### 2.2 Simulation

Lower resolution numerical simulation based on the Bloch equations [9] was performed using in-house software [10] that directly converts a Pulseq sequence into a list of commands. These commands in turn are applied to individual isochromats, defined by their proton density, location in 3D space, and T_1_/T_2_ relaxation times, that make up a numerical phantom. When exposed to temporally and spatially varying magnetic fields such as those defined by a pulse sequence program, the isochromat's magnetization vector evolves according to its initial condition, innate parameters, and the external driving fields. A numerical library is used to solve the differential equations. In the end, the detectable signals from the transverse magnetization are added up across all isochromats in a phantom to generate the raw MRI signal.

All simulations were performed on a Windows 10 operating system with an Intel(R) Core i7–8650 U CPU. Specific parameters used for the simulation were: FOV = 250 mm, slice thickness = 5 mm; TR = 4500 ms, TI = 200 ms, and TE = 10 ms for IRSE; TR = 4500 ms and TE = 10 ms for TSE.

### 2.3 Acquisition and reconstruction

Experiment parameters are shown in Table 1. We reconstructed the raw data with simple 2D Inverse Fast Fourier Transform on each channel after correcting the ordering in the phase encoding dimension. The channels were combined using sum-of-squares. For the quantitative mapping experiments, each k-space was first multiplied with a 128-point 2D Hamming filter [11] before reconstruction to reduce ringing artifacts around and inside the small T_1_/T_2_ spheres.

### 2.4 Quantitative mapping and image metrics

For each mapping experiment, the corresponding signal equation (T_1_: $S=A(1-2\exp\left(-TI/T_{1}\right)+\exp\left(-TR/T_{1}\right))+B$); T_2_: $S=A\exp(-TE/T_{2})+B$; A: signal scaling; B: signal offset value) was fitted to the multi-contrast images voxel-by-voxel using the MATLAB curve fitting toolbox (see scripts in the analysis data folders) within a manually selected overall ROI that covers the circular phantom. Following generation of T_1_/T_2_ maps, small sphere-wise ROIs were manually selected to ensure only interior voxels are used. For each ROI, the mean and standard deviation of T_1_/T_2_ values were computed.

For the qualitative images, Peak Signal-to-Noise Ratio (PSNR) and Structural Similarity Index Measure (SSIM) [12] were computed against equivalent vendor images acquired in the same session. Normalization was performed across the 11 slices for each set before comparison. The seven standard ACR tests were performed on the corresponding slices according to the published guidance [5].

### 2.5 Safety

The open-source sar4seq library was used to generate predicted time-averaged RF power and Specific Absorption Rate (SAR) for each sequence [13,14]. At acquisition, values were also read off from vendor software for measured SAR, time-averaged RF power, and percent Peripheral Nerve Stimulation (PNS) threshold. All evaluation assumed a 70 kg subject.

## Ethics Statements

Our work did not involve human or other animal subjects and adheres to ethics in publishing standards.

## CRediT authorship contribution statement

**Gehua Tong:** Formal analysis, Investigation, Methodology, Software, Visualization, Writing – original draft, Writing – review & editing. **Andreia S. Gaspar:** Investigation, Validation, Visualization. **Enlin Qian:** Methodology, Software. **Keerthi Sravan Ravi:** Software. **John Thomas Vaughan:** Writing – review & editing. **Rita G. Nunes:** Funding acquisition, Project administration, Resources, Supervision, Validation, Visualization. **Sairam Geethanath:** Conceptualization, Funding acquisition, Methodology, Project administration, Resources, Software, Supervision, Writing – review & editing.

## Declaration of Competing Interest

The authors declare that they have no known competing financial interests or personal relationships that could have appeared to influence the work reported in this paper.

## Data Availability

Open source pulse sequence validation framework (Original data) (Mendeley Data).
